# Mammary Analogue Secretory Carcinoma (MASC) of salivary gland in four Mexican patients

**DOI:** 10.4317/medoral.19874

**Published:** 2014-09-30

**Authors:** Mónica L. Serrano-Arévalo, Adalberto Mosqueda-Taylor, Hugo Domínguez-Malagón, Michal Michal

**Affiliations:** 1 Departamento de Citopatología, Instituto Nacional de Cancerología, México, D.F; 2 Departamento de Atención a la Salud, Universidad Autónoma Metropolitana Xochimilco, México, D.F; 3 Departamento de Patología, Instituto Nacional de Cancerología, México, D.F; 4 Sikl’s Department of Pathology, Faculty of Medicine in Pilsen, Czech Republic

## Abstract

The Clinco-pathological, immunohistochemical and molecular findings of four cases of Mammary Analogue Secretory Carcinoma (MASC) of salivary glands found in Mexico are described. The cases were extracted from 253 salivary gland tumors from a single institution in Mexico City. The 85 candidates for initial selection were: low grade mucoepidermoid carcinoma (MEC) (N=70 ), acinic cell cancinoma (AciCC) (N=14), papillary cystadenocarcinoma (N=1), and adenocarcinoma NOS (N=0). Tumors with some histological features consistent with MASC (N= 17, 6.7%) were studied by immunohistochemistry for mammaglobin, STAT5, and S-100 protein and four cases were positive (1.5%), thus the diagnosis of MASC was established, and these were submitted for molecular studies for *ETV6-NTRK3*. Fusion gene was demonstrated in three cases, two had been erroneously diagnosed as poorly granulated AciCC, and one as low grade MEC with microcystic pattern. Female gender predominated (3:1); one occurred in the parotid, two in minor salivary glands and one in the submaxillary gland; infiltrating borders, atypical mitosis and lymph node metastases were seen in the parotideal tumor. Two patients with major salivary gland tumors are alive and well at 10 and 20 months respectively, the two patients with minor salivary gland tumors are lost. It can be concluded that is important to think in MASC in poorly granulated AciCC and low grade MEC with microcystic pattern. Immunohistochemisty studies confirm the diagnosis, preferentially supported by molecular studies. MASC may follow aggressive behavior or transform into a high grade neoplasm.

** Key words:**Acinic cell carcinoma, ETV6-NTRK3, Mammary Analogue Secretory Carcinoma, secretory breast carcinoma.

## Introduction

Mammary Analogue Secretory Carcinoma (MASC) of salivary glands was described by Skalova *et al*. in 2010 (1). It is a neoplasm that shares the same histological appearance and molecular alterations of secretory breast carcinoma. Most cases have an specific translocation t(12;15) (p13; q25) originating fusion of genes *ETV6-NTRK3* that codifies a chimeric tyrosine kinase. This genetic rearrangement has also been found in congenital fibrosarcoma, mesoblastic nephroma and acute myeloid leukemia (2). MASC has been reported as a low grade tumor with microcystic, cystic-papillary, glandular, and solid patterns. The cells have bland nuclei, eosinophylic granular or vacuolated cytoplasm, and show intraluminal or intracytoplasmic secretion (1). Recent studies have demonstrated that MASC can bear a resemblance to other salivary gland tumors such as adenocarcinoma NOS, mucoepidermoid carcinoma (MEC), cystadenocarcinoma, and poorly granulated acinic cell carcinoma (AciCC) (1,3). According to Chiosea *et al*. (3,4), MASC has a male predilection, and predominantly involves the parotid gland as a mass growing in 2 to 36 months.

In Mexico this entity has not been reported, therefore a retrospective study was undertaken to identify cases of MASC by immunohistochemistry, and support the diagnosis with genetic studies.

## Material and Methods

Cases of MASC were searched among 253 salivary gland tumors from the files of the Pathology Department of the Instituto Nacional de Cancerologia at Mexico City, from January 2006 to November 2012. The protocol was approved by the Institution Review Board.

Initially all cases with diagnosis of low grade MEC, AciCC, papillary cystadenocarcinoma, and adenocarcinoma NOS were considered for histological review. From this initial group (N=85), the following features were searched: macrocystic, microcystic or papillary patterns, pale eosinophylic, granular or vacuolated cytoplasm, glassy secretion and intracellular mucin. Cases with at least three of these features (N = 17) were selected and studied by immunohistochemistry for STAT5, mammglobin, and S100, and the tumors that expressed the three antibodies were finally selected for molecular studies for *ETV6-NRK3* fusion gene. Clinical information of these final cases included: evolution, gender, tumor site and age of presentation. Morphological characteristics considered were: tumor size, macroscopic appearance, predominant pattern (cystic, papillary, cribriform and solid), growth (encapsulated, demarcated or invasive), extraglandular invasion, cytoplasmic vacuolation, intra cytoplasmic secretion, nuclear grade, prominence of nucleolus, mitosis per 10 HPF, necrosis, perineural invasion and vascular permeation.

Immunohistochemistry

For immunohistochemical studies, 4-μm-thick sections were cut from paraffin blocks, mounted on slides coated with 3-aminopropyltriethoxy-silane (Sigma, St. Louis, USA), deparaffinized in xylene, and rehydrated in descending grades (100% to 70%) of ethanol. Sections were then subjected to heat-induced epitope retrieval by immersion in a CC1 solution at pH 8, at 95°C. Endogenous peroxidase was blocked by a 5-minute treatment with 3% hydrogen peroxide in absolute methanol. The slides were then stained by immunostainer Bench Mark ULTRA (Roche). The bound antibodies were visualized using the Histofine Simple Stain MAX PO (Multi) Universal Immuno-peroxidase Polymer, Anti-Mouse and Rabbit (Nichirei Biosciences inc., Tokyo, Japan), and 3-3´-diaminobenzidine (Sigma) as chromogen. The slides were counterstained with Mayer’s hematoxylin. Appropriate positive and negative controls were employed. The following primary antibodies were used: STAT5 (polyclonal, Enzo, 1:200), S-100 protein (polyclonal, Dako, 1:300), mammaglobin (clone 304-1A5, Dako, 1:300).

Molecular studies

RNA from the formalin fixed paraffin embedded tissue was extracted using the RecoverAll Total Nucleic Acid Isolation Kit (Ambion, Austin, TX, USA). cDNA was synthesized using the Transcriptor First Strand cDNA Synthesis Kit (RNA input 1 μg) (Roche Diagnostics, Mannheim, Germany). All procedures were performed according to the manufacturer’s protocols. Amplification of a 105-bp product of the 2-microglobulin gene, 126-bp product of the PBGD gene and 247 bp product of PGK gene was used to test the quality of the extracted RNA as previously described (5-7). A detection of 110 bp fragment of *ETV6-NTRK3* fusion transcript was performed according to the method described by Bourgeois *et al*. (8).

Briefly, two of cDNA was added to reaction consisted of 12.5 µl of Hot Start Taq PCR Master Mix (QIAgen, Hilden, Germany), 10 pmol of each primer (TRKC1059 complementary to NTRK3 with sequence CAGTTCTCGCTTCAGCACGATG and TEL971 complementary to ETV6 with sequence ACCACATCATGGTCTCTGTCTCCC) and distilled water up to 25 µl. The amplification program comprised of denaturation at 95°C for 14 minutes and then 45 cycles of denaturation at 95°C for 1 minute, annealing at 65°C for 1 minute and extension at 72°C for 1 minute. The program was finished by incubation at 72°C for 7 minutes.

Successfully amplified PCR products of the *ETV6-NTRK3* fusion gene were purified with a Montage PCR Centrifugal Filter Devices (Millipore, Billerica, USA). Then, PCR products were both sides sequenced using a Big Dye Terminator Sequencing kit (Applied Biosystems, Foster City, USA), run on an automated genetic analyzer ABI Prism 3130xl (Applied Biosystems) at a constant voltage of 13.2 kV for 20 minutes and compared to the Gene Bank sequence.

## Results

Of the 253 salivary gland tumors, 85 tumors with the diagnoses of low grade MEC (N = 70), AciCC (N = 14), papillary cystadenocarcinoma (N = 1), were considered for initial search; from these 85 cases, 17 were considered morphologically consistent with MASC (6.7 %) and studied by immunohistochemitry. Four of these cases (1.5 % of all) were positive for S-100 protein, mammaglobin and STAT5, thus the diagnosis of MASC was established. Molecular study demonstrated *ETV6-NTRK3* fusion gene in three, and was negative in one that was still considered as MASC because of the positive result of the immunohistochemistry.

-Clinical findings:

 Table 1 shows that the four patients complained of increase of volume lasting from 2 months to 4 years. There were three women and one man aged 28-83 years. The tumors measured 5 to 75mm, three cases were located outside the parotid, one in the submaxillary gland, and two affecting minor glands of the oral mucosa. Treatment included excisional biopsy or wide resection. The parotid tumor (case 1) was regarded as high-grade, and treated by parotidectomy and regional lymphadenectomy, it presented lymph node metastasis and received adjuvant radiotherapy. On follow-up, the two cases located in major glands were alive and well at 10 and 20 months respectively; the two remaining cases were lost to follow up.

**Table 1:**
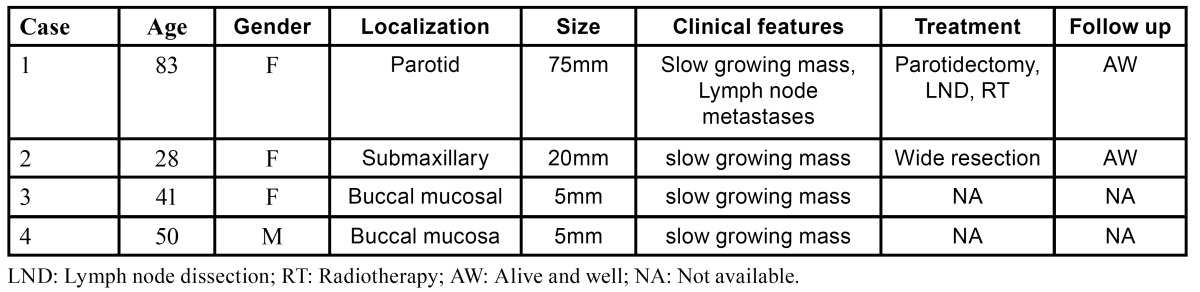
Clinicopathological features of the four cases of MASC in the present series.

-Morphological findings.

The parotid tumor disclosed on cut surface pale solid, lobulated, granular, and cystic areas, surrounded by irregular borders. Macroscopic features of the other three cases are unavailable.

**Figure 1:**
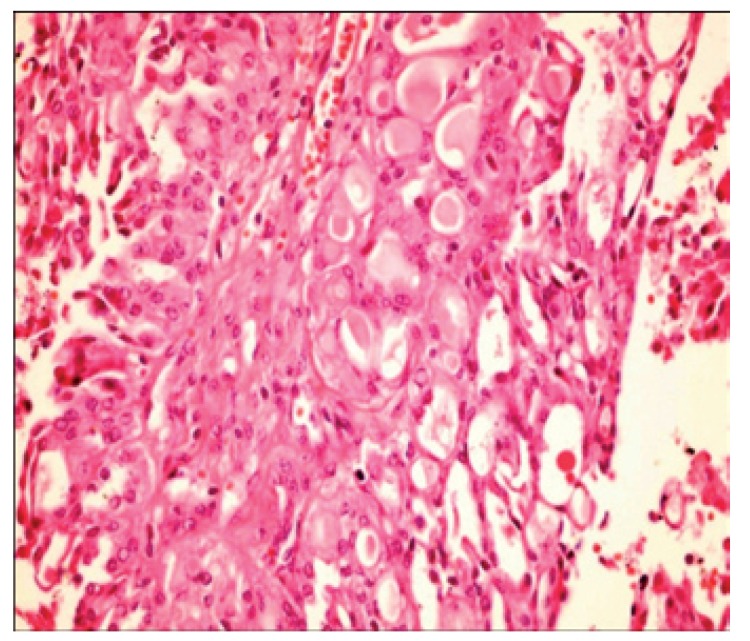
Microcystic pattern.

**Figure 2:**
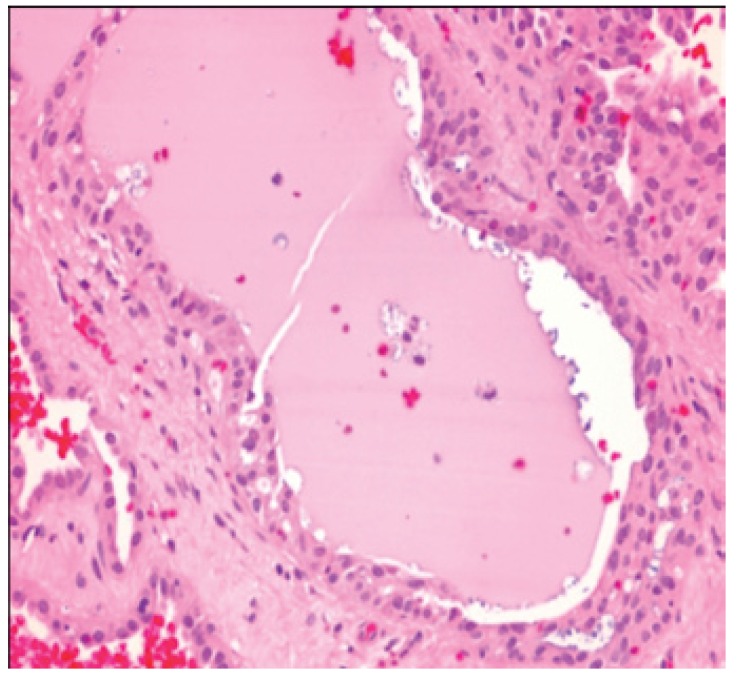
Macrocystic pattern with intraluminal secretion.

**Figure 3:**
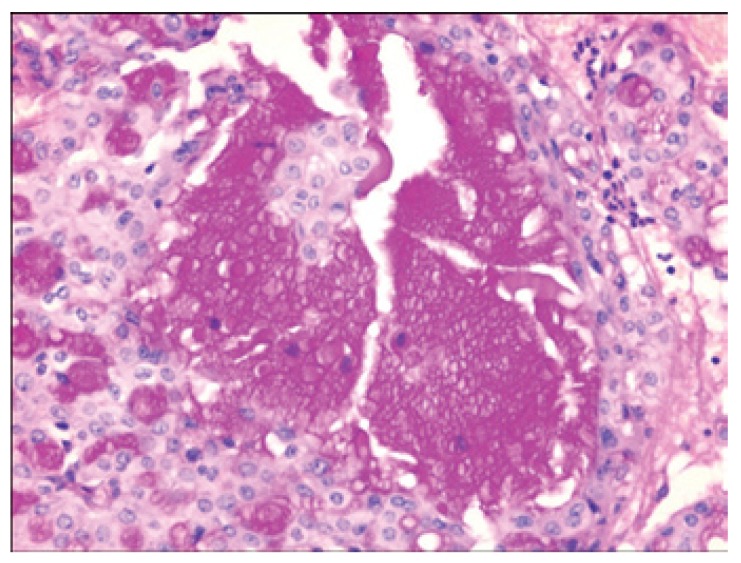
Intraluminal and intracytoplasmic PAS-D positive secretion.

**Figure 4:**
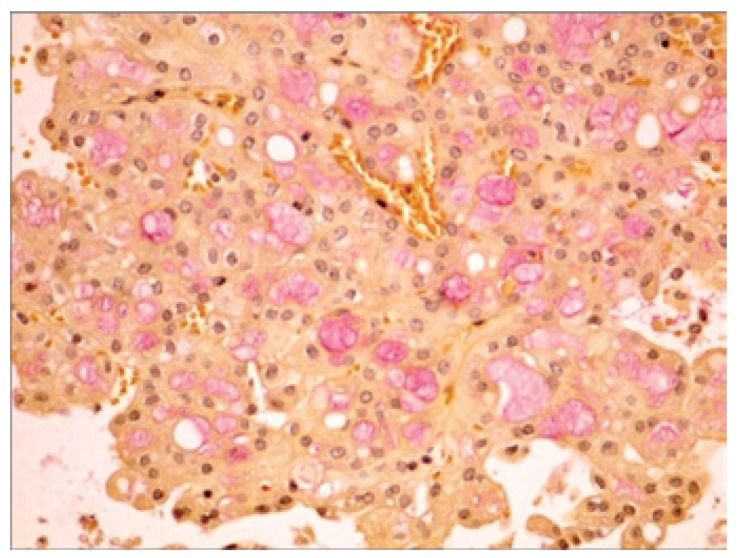
Mucicarmine positive cells.

**Figure 5:**
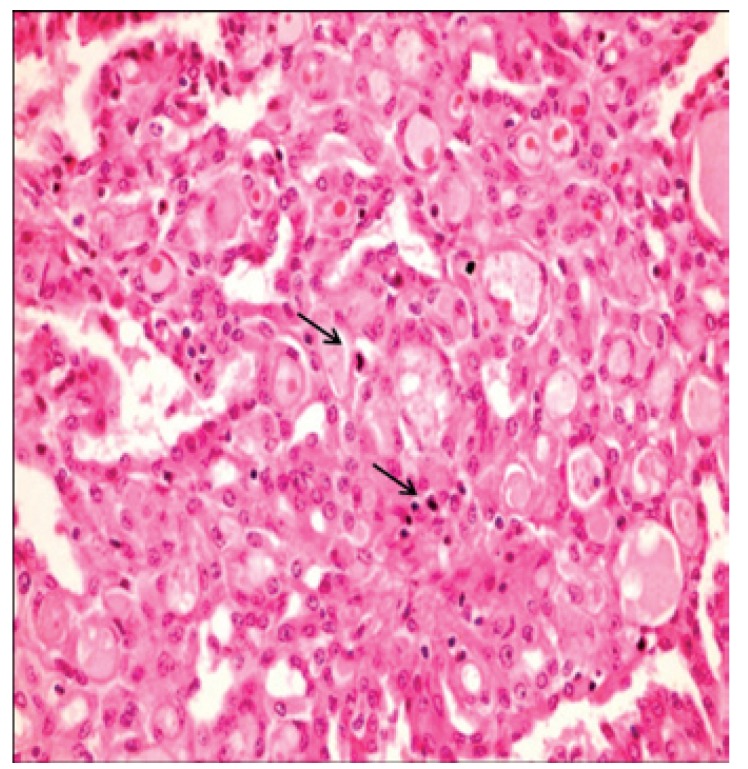
Atypical mitosis.

**Figure 6:**
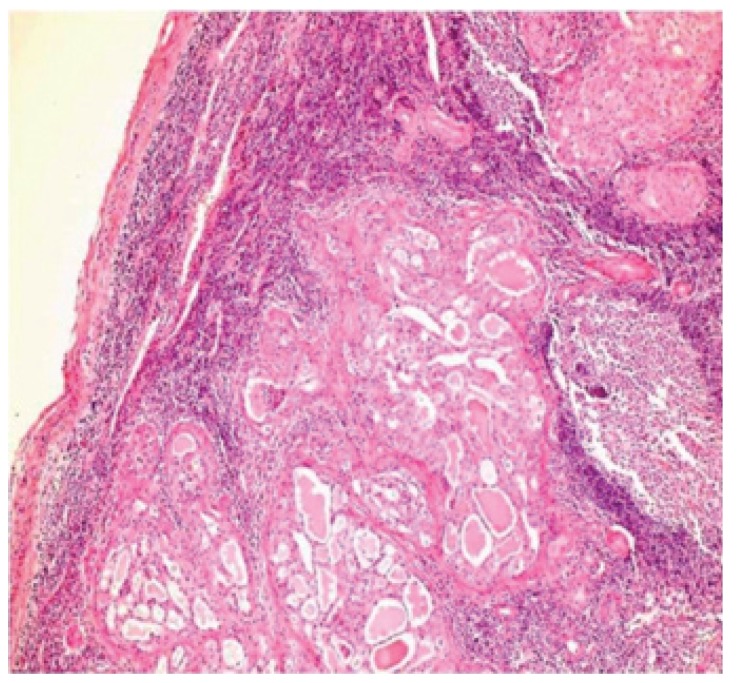
Lymph node metastasis of MASC.

 Table 2 shows the salient histopathological features. All cases have infiltrating borders with predominant microcystic pattern, followed by papillary and macrocystic patterns (Figs. 1,2). Solid areas were seen focally in 2 cases and no extraglandular invasion was seen. Cells with pale eosinophylic, granular or vacuolated cytoplasm and cribriform structures with glassy secretion and intracellular mucin that was positive for mucicarmine and PAS/D were found in all cases (Figs. 3,4). Cases 2, 3 and 4 had a low nuclear grade with round or ovoid uniform nuclei with visible nucleoli. An average of one mitosis in 10 high power fields was found (Fig. 5). Case 1 metastasized to lymph node, showed high nuclear grade clumped chromatin, and 5 mitosis per 10 HPF (Fig. 6). No necrosis, perineural or vascular permeation was found.

**Table 2:**
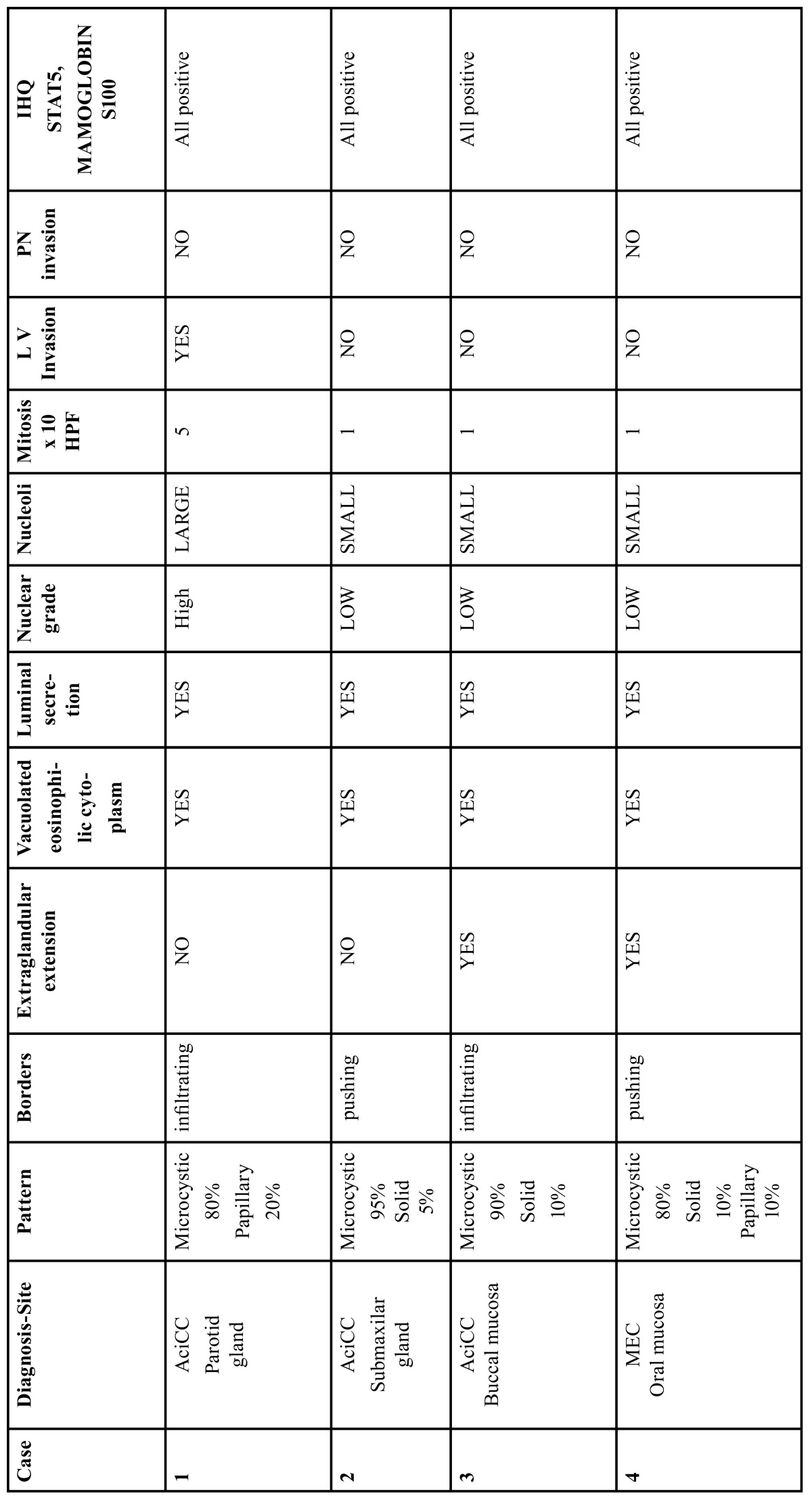
Morphological features.

-Immunohistochemical findings.

**Figure 7:**
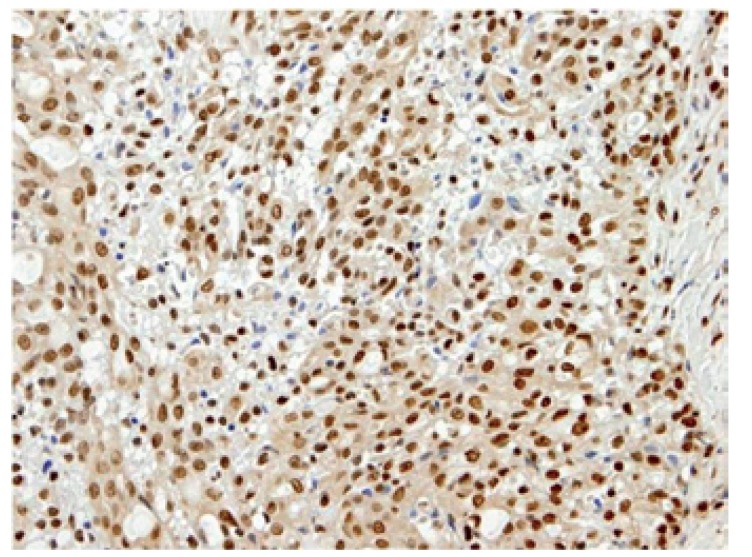
STAT5 show strong nuclear expression in tumor cells.

**Figure 8:**
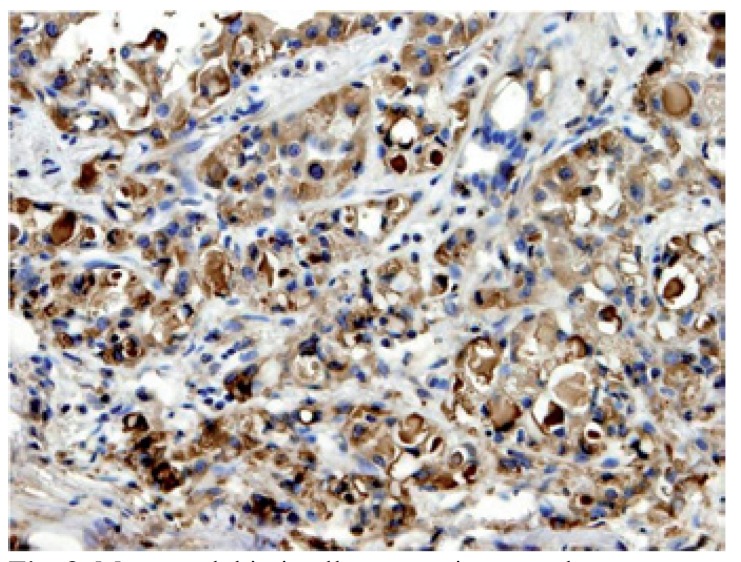
Mammaglobin in all cases stains cytoplasm.

The four final cases were positive for S-100 protein, mammaglobin and STAT5 (Figs. 7,8); Cases 1 and 3 were erroneously diagnosed as poorly granulated ACC, case 4 as low grade MEC, and case 2 suspected as MASC.

-Molecular findings

The four cases of MASC were studied by molecular genetic methods. In all of them the quality of extracted RNA was good enough for subsequent detection of *ETV6-NTRK3* fusion gene, at least two control genes were amplified. In 3 samples taken from 3 positive control cases, RNA was analyzable, too. The analysis of *ETV6-NTRK3* fusion transcript revealed positivity in Cases 1, 3 and 4 but was negative in case 2. Positive controls of known MASC were employed. For negative controls various tumors of salivary gland origin with undetermined secretory features were used.

## Discussion

MASC was recently described by Skalova *et al*. (1) in a series of 16 salivary gland tumors, in which they featured histological and immunohistochemical characteristics identical to breast secretory carcinoma. These tumors were lobulated, displayed macro and microcysts, and less frequently were solid and papillary. Connor *et al*. (9) found that the majority of tumors with microcystic structures tended to be less invasive than those with solid architecture that often showed extraglandular invasion. The neoplastic cells have pale, ovoid nuclei with minimal pleomorphism, the cytoplasm is vacuolated or eosinophilic; intracytoplasmic secretion with a “glassy” appearance positive for PAS/D is found within cystic spaces. The proliferative index as determined by Ki67 immunoexpression is low, perineurial invasion may be found and no necrosis is observed. By immunohistochemistry the tumor cells are positive for S100, STAT5 and mamoglobin, wich are considered as diagnostic criteria for this entity (9). The neoplastic cells also express CK7, CK8, CK18, CK19, GCDFP15, and EMA but they are non specific and other salivary gland tumors can be also positive. The immunological profile suggests origin of MASC from striated duct, which differs from AciCC that originates in the acinic and intercalated ducts (9) expressing lactoferrin, alpha 1-antitrypsin, alpha 1-antichymotrypsin, and carcinoembryonic antigen (10).

In studies of breast tumors by Reis-Filho *et al*. (11) and 

Pia Foschini *et al*. (12) the authors found absence of ETV6 rearrangement in AciCC, which is in agreement with the notion that secretory carcinoma and AciCC in mammary gland are different entities.

The translocation *ETV6-NRK3* is not completely specific of breast secretory carcinoma and MASC; it is also present in congenital fibrosarcoma, mesoblastic nephroma, and acute myeloid leukemia (2). The biologic significance of the translocation is fusion of the transcriptional regulator gene *ETV6* with the membrane receptor kinase-type *NTRK3* activating cell proliferation and survival (2). However, a negative molecular study does not necessarily exclude the diagnosis of MASC (1,9), immunohistochemical studies positive for STAT5, mammoglobin and S100 are diagnostic. The molecular recognition of MASC may be important in future because tyrosine–kinase inhibition is becoming a viable therapeutic option (3).

MASC has been reported as more frequent in males, however in this small series the opposite was true. No racial predilection is informed. Although the neoplasm was first described in occidental countries, in a study by Jung et al. (13), 13 cases of salivary gland tumors with the *ETV6* translocation in Orientals were reported. The clinical presentation is as a non-tender mass, growing in a few months to several years and it is reported as more frequent in the parotid gland (1,12); however, in the paper by Chiosea *et al*. (3) a higher number of lesions in minor salivary glands were found similar to the present study. Another report of MASC in minor salivary glands was published by Bishop *et al*. (14), in which the authors state that almost all extraparotid AciCC corresponded to MASC; the diagnosis of AciCC requires evidence of zymogen granules, and in the absence of this key finding the diagnosis of MASC should be considered (13).

The disease free period reported in the literature for MASC is 71 to 115 months, which is slightly shorter than AciCC, which ranges from 92 to 148 months (3).

MASC has a slightly higher risk for regional lymph node involvement than AciCC (3), it may follow an aggressive behavior or transform into a high grade neoplasm. Skalova reported 3 cases with conventional MASC and a population of anaplastic cells with perineural invasion (15).

Postoperative treatment is not standardized because the rarity of the entity; radiotherapy and chemotherapy have been used empirically.

Differential diagnosis include: low-grade MEC which is characterized by the presence of various cell types, it may contain variable proportions of squamous cells, clear cells, mucocytes, oncocytes, intermediate cells, and columnar cells, and also may show a sclerotic fibrous stroma or extravasation of mucin. Cystadenocarcinoma may resemble cribriform ductal carcinoma in situ (DCIS) not observed in MASC. AciCC presents acinic cells, intercalated duct cells or clear cells, and their cytoplasm is rich in zymogen granules. The granule-poor variant of AciCC (3) is difficult to differentiate from MASC, it is negative for S-100 protein, STAT5 and mammaglobin, and positive for transferrin, lactoferrin, alpha 1-antitrypsin, alpha 1-antichymotrypsin carcinoembryonic antigen, and Leu M1 antigen (10).

In conclusion, in this paper 4 cases of MASC diagnosed in Mexico are presented, three of which were erroneously diagnosed as other type of salivary gland neoplasms. It is important to consider MASC in salivary gland tumors with with microcystic pattern, composed of cells with pale eosinophylic, granular or vacuolated cytoplasm that have glassy secretion and intracellular mucin. Low-grade MEC, poorly granulated AciCC, papillary cystadenocarcinoma and adenocarcinoma NOS have to be studied for this posibility. Immunohistochemisty studies are necessary to confirm the diagnosis, preferentially supported by molecular studies.
